# Dietary Behavioural Preferences of Spanish and German Adults and Their Translation to the Dietary Recommendations of a Personalised Nutrition App in the Framework of the Stance4Health Project

**DOI:** 10.3390/nu17050912

**Published:** 2025-03-06

**Authors:** Daniel Hinojosa-Nogueira, Beatriz Navajas-Porras, Silvia Pastoriza, Adriana Delgado-Osorio, Ángela Toledano-Marín, Sascha Rohn, José Ángel Rufián-Henares, José Javier Quesada-Granados

**Affiliations:** 1 Department of Nutrition and Food Sciences, Institute of Nutrition and Food Technology, Biomedical Research Centre, Universidad de Granada, 18071 Granada, Spain; dhinojosa@ugr.es (D.H.-N.); beatriznavajas@ugr.es (B.N.-P.); spdelacueva@ugr.es (S.P.); adrianadelgado@ugr.es (A.D.-O.); antolemarin@correo.ugr.es (Á.T.-M.); quesadag@ugr.es (J.J.Q.-G.); 2 Department of Food Chemistry and Analysis, Institute of Food Technology and Food Chemistry, Technische Universität Berlin, 13355 Berlin, Germany; rohn@tu-berlin.de

**Keywords:** food choice, portion size assessment, meal intake behaviour, personalised nutrition

## Abstract

**Background/Objectives**: The influence of individual differences in the selection of food portions can have a deep effect on recommendations for personalised nutrition. In addition to typical aspects such us energy density and nutrient composition, portion size is important for dietary recommendations. This study examined the dietary behaviours and portion size selection of 224 subjects in Spain and Germany to use such information to improve dietary adherence to a personalised nutrition app. **Methods**: An online questionnaire administered to adults in Spain and Germany collected sociodemographic data and dietary habits. The measurement of portion sizes was derived from a classification ranging from XXS to XL across 22 food groups, with assistance from a photographic atlas. **Results**: Significant differences across dimensions were found. Dietary habits showed that omnivores were the majority in both countries, with significant differences in the consumption of bread, desserts, and beverages. The Mediterranean diet was significantly followed by the Spanish group, reflecting cultural differences. Body mass index (BMI) was slightly higher among Germans, although both populations fell within the normal ranges. Portion size comparisons revealed statistically significant differences in the consumption of various food items between the two countries. Spaniards consumed higher amounts of rice, meat, and legumes, while Germans consumed larger portions of stews, lasagne, and pizza. These variations highlight differing dietary habits influenced by cultural preferences and dietary guidelines. **Conclusions**: The findings support the development of novel personalised nutrition apps that consider user preferences and enhance dietary adherence, thereby contributing to improved dietary recommendations and health outcomes.

## 1. Introduction

Dietary choices are influenced by a variety of factors, including personal preferences, lifestyle, environment, socioeconomic status, and religious, or philosophical beliefs. These factors influence people’s dietary behaviours [1]. Understanding dietary habits through the identification of factors influencing food selection and the dietary status of individual behaviour is therefore vital [2]. However, maintaining an adequate nutritional status can be complicated. Dietary recommendations are often based on guidelines for large groups of healthy people. Consequently, these recommendations are very general and frequently ignore that different individuals have a wide range of responses to different dietary patterns [3,4]. The heterogeneous nutritional responses of individuals increase the need for the development of precision or personalised nutrition (PN). In recent years, advances in nutrition and food sciences have aided in the analysis and comprehension of the specific needs and dietary behaviours of each individual [5,6].

PN has great potential, particularly when combined with the power and accessibility of new technologies [7]. New dietary evaluation tools are being developed and validated using web-based tools [8]. Some examples are ASA-24 [9], or INTAKE24, which introduces additional features such as home measurements or pictures of food rations [8]. Mobile applications (apps) have been instrumental in facilitating the accurate estimation of the nutritional value of consumed foods, dietary habits, and food intake, thereby contributing to the enhancement of public health [6]. Currently, it is estimated that there are around 10,000 apps available for nutrition and diet [10]. Speak4Diet is an example of how a mobile application can use technology to empower people to improve their diet by integrating this technology into their daily lives [11]. Apps offer greater versatility and faster feedback, making them a more cost-effective option for improving healthy eating habits [12]. Therefore, apps enable rapid data analysis, which could help improve access to quality nutritional data [13].

In recent years, a proliferation of food and drink options has been observed, precipitating substantial shifts in consumption patterns, encompassing both quality and quantity [2]. As a result, making appropriate food choices is also linked to portion size control [14]. Food portion size is traditionally measured in terms of weight or volume. Although weight can be determined accurately using a scale, it is time-consuming and requires a high level of motivation from consumers [15]. More convenient would be a fast visualisation method. Several different visual methods are commonly used to estimate food consumption, such as the use of household measurements, where common objects are used to relate their size to the portions consumed [16]. Identical artificial figures of food have also been used [17]. The use of 3D-printing technology has made it more cost-effective for estimating the portion size using 3D-models [2]. Another tool for estimating food portion sizes is the use of standardised photos of food portions. This is a cost-effective and portable method that is widely considered a useful tool for estimating food portion sizes. These photos are grouped into a digital photographic food atlas [2,16,17]. Currently, thanks to new technologies, food portion estimations are continuously improving. For instance, image recognition combined with bioinformatics techniques can provide accurate estimations [6,18]. Other examples could include infrared technology or thermal cameras, which are rapidly evolving [18].

Furthermore, a multitude of additional factors have the capacity to influence dietary decisions in relation to the consumption of particular food items, the size of portions consumed, and also to affect other dietary habits [1]. Nevertheless, the issue of portion sizes remains a significant challenge in estimating and formulating dietary recommendations. In the context of PN, the development of apps to monitor, control, and manage diets has emerged as a significant advancement. However, to ensure the effective implementation of these apps, it is crucial to obtain and use precise estimations of portion sizes.

Based on this evidence, the aim of the present study was to investigate the dietary behaviour of adults from two of the countries (Germany and Spain) involved in the European research project Stance4Health [19]. The results of this study would help to understand and develop more specific tools to help with portion size recommendations and other dietary aspects, in order to improve the accuracy and suitability of the i-Diet app [20], which is the main focus of the aforementioned project.

## 2. Materials and Methods

### 2.1. Study Participants

This work is part of the Stance4Health project (https://stance4health.com/, accessed on 19 January 2025). The participants were recruited from Spain and Germany, where nutritional interventions were planned to be carried out as part of the overall project goal. No inclusion or exclusion criteria were applied, and the participants solely comprised adults from the general population, aged 18–82 years. The determination of sample size was based on a correlation-based power analysis expecting an effect size of *ρ* = 0.35, with an α error probability of 0.05 and a desired statistical power (1 − *β*) of 0.95. With a target population of 200 participants per country, as established in the European S4H project, the finite population correction was applied [21], resulting in a need to recruit approximately 66 people per country. This strategy was developed to optimise the balance between logistical feasibility and the desired accuracy in detecting changes in consumption habits in the Spanish and German populations, serving as a foundation for the subsequent personalised nutrition study. The recruitment process involved an online questionnaire, which was distributed through social media and via the networks of the researchers in charge per country. The study was approved by the Ethics Committee of the University of Granada (protocol code 1080/CEIH/2020) and all subjects voluntarily participated in the study. The data were processed as specified in Regulation (EU) 2016/679 “General Data Protection Regulation (GDPR)”, and the Declaration of Helsinki on research ethics was followed. To ensure participant anonymity, the collected data were anonymised.

### 2.2. Dietary Behaviours and Questionnaire Construction

The developed questionnaire included personal characteristics such as age, weight, height, and socioeconomic information. Body Mass Index (BMI) was calculated using the formula (kg/m^2^). In addition, the questionnaire asked about dietary behaviours such as eating alone or eating bread with meals or drinking and the type of drinks consumed, among other relevant information. To determine whether the dietary patterns were similar between both populations, the 13-questions questionnaire on adherence to the Mediterranean diet (AMD) was also used, which was a modified version that does not consider alcoholic beverages [22]. Good AMD was defined as a score of 8 points or more [22]. The questionnaire was developed and provided online through Google Forms.

The creation of the questions regarding portion size was conducted through the integration of diverse tools, including food frequency questionnaires, food consumption patterns in Spain and Germany, data from the photographic food atlas, and culinary techniques and portion sizes detailed in recipes [16,23,24,25,26,27,28,29,30,31]. The standard food sizes were defined according to the methodology established in some photographic food atlases [15,32]. In order to standardise portion sizes, six different images were used. The food photographs were obtained from a previously validated photographic food atlas [17,33,34,35].

Food portions were presented from left to right in the order of size, from small to large, or in the order of weight, from light to heavy. The sizes were assigned ranging from XXS, XS, S, M, L, and XL, obtaining a more understandable methodology for the subjects [36]. Food selection was generalised for the choice of food photos to estimate equivalent food. The study summarised and classified the consumption size images of different food groups and dishes in diverse conditions and with various tableware into 22 categories based on food type and size (Table 1). For instance, a salad with green leaves was used as an example, which can be applied to different types of salads. This allows users to select a portion size for each category according to their preferences.

### 2.3. Data Statistical Analysis

The collected data comprised both quantitative and qualitative variables. Data were presented as percentages or as means and standard deviations. To explore these categorical variables, specific statistical analyses were used. Tests of association, such as the Chi-square test, were used to assess the relationship between variables. In specific cases, categorical variables were coded, such as part of the MANOVA analysis, which was used to examine differences in multivariate proportions to determine if there were significant differences between groups. For quantitative variables, the Kolmogorov–Smirnov test was used to analyse the data, followed by Spearman correlations to assess the associations between the different variables. The statistical differences between the groups were evaluated according to the Mann–Whitney U and Kruskal–Wallis test. The SPSS 26.0 statistical software was used to analyse the data. The level of significance was set at *p* < 0.05. Graphics were generated using the Python 3.7 module. The calculation of statistical power was carried out using G*Power software (version 3.1.9.7).

## 3. Results

### 3.1. Characteristics of the Subjects and Dietary Habits

The study comprised a total of 224 participants, with ages ranging from 18 to 80 years in Spain and from 20 to 82 years in Germany (Table 2). The average age was 35.4 years. Of the participants, 38% were Spanish and 62% were from Germany. The study included 77 men and 147 women, 97.3% of whom were European (Spanish or German nationality) and the remaining nationalities residing in Spain or Germany. Of the participants, 18% reported allergies or food intolerances. The most common allergies were towards dairy products and nuts. Some allergies to fruit peel and plant lipid transfer proteins (LTP), or intolerances to additives were also identified among the participants.

A total of 81.3% of surveyed participants completed higher education and 15.6% declared having only secondary education. For the rest, primary education studies were declared. Of those surveyed, 59.8% were employed, with 11.6% working part-time. A total of 36% of the participants were studying, while only 3.6% and 0.9% were retired or unemployed, respectively. The survey results show that 52.7% of the participants were single. Additionally, 37.5% of the participants lived with their family, 28.1% with their partner, and 15.6% lived alone, while 18.3% declared that they lived with friends. In terms of housing, 49.5% of the participants lived in rented accommodation while 42% owned their own home. Furthermore, 74.9% of the participants reported living in urban areas, while the remaining participants reported living in rural areas. In total, 10% of the total population were vegan or vegetarian (all from Germany).

**Figure 1:**
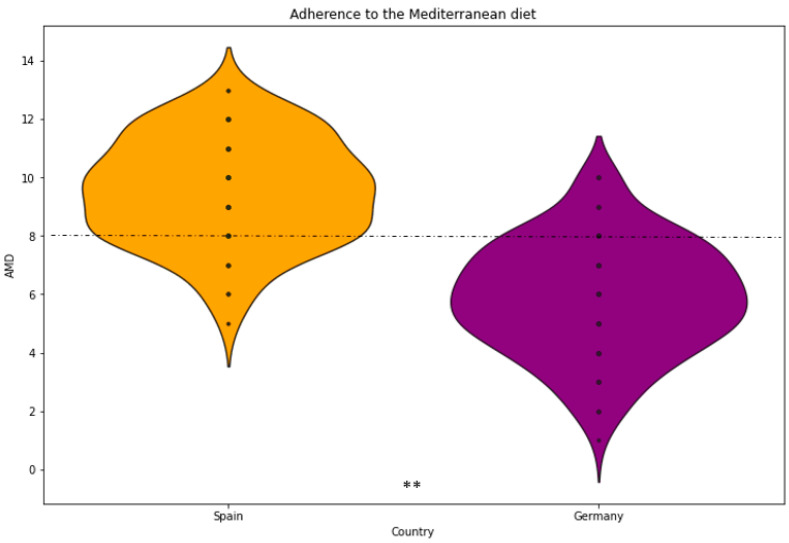
Adherence to the Mediterranean diet (AMD) by country. A score of 8 or higher indicates high AMD. Differences between Spanish and German samples. ** Significant at the *p* < 0.05 level.

**Figure 2:**
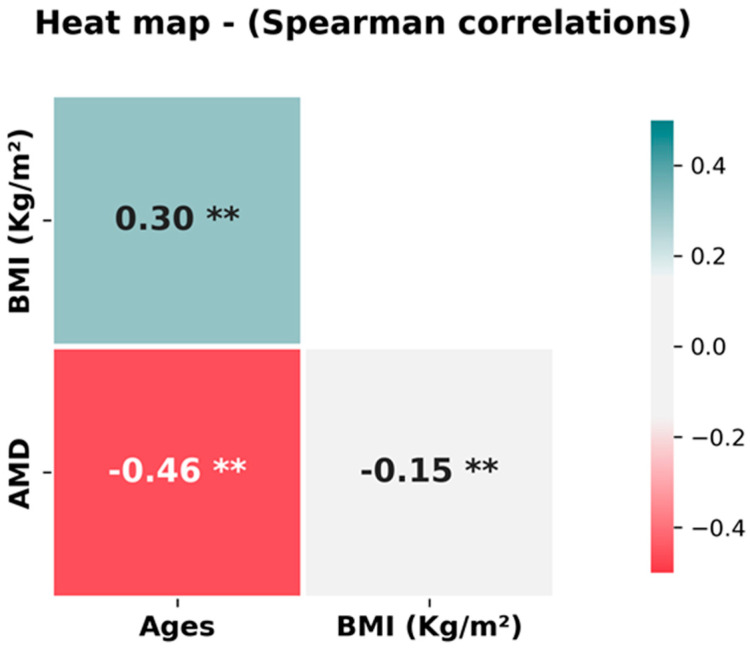
Heat map showing significant Spearman correlations between BMI, age, and adherence to the Mediterranean diet (AMD). ** Correlation significant at *p* < 0.05 level.

Regarding dietary behaviour, 72% of the participants ate in company. In general, 89.7% used spices, which was significantly higher in the Spanish cohort. Furthermore, 84.4% of the participants preferred a single dish. Significant differences were observed between countries, with only 28% of participants in Spain choosing other options, such as a menu with several dishes, compared to 9% in Germany. In total, 62% of participants eat desserts daily or regularly. In addition, 44% of participants did not consume bread during meals, while close to 95% consumed some kind of drink during meals. When examining data by country, it is interesting to note that in Spain, 81% of participants drank water and only 19% drank other beverages. In Germany, this percentage changes, with 54% drinking water, 29% drinking sparkling water, and the rest reporting other drinks. Related to lifestyle, 76% reported engaging in light or moderate physical activity. On average, the participants had a height of 171 cm and a weight of 70.7 kg, with a mean BMI of 23.8 ± 4.3 (kg/m^2^). Statistically significant differences were found between BMI in men (24.8 kg/m^2^) and women (23.3 kg/m^2^) (*p*-value: 0.008). The mean score for adherence to the Mediterranean diet (AMD) was 7.4 ± 2.5, indicating generally low adherence (especially among German respondents). Statistically significant differences were found between the AMD of the Spanish and German populations (Figure 1) (*p*-value: 0.001). Significant correlations between BMI, age, and AMD were found (Figure 2), suggesting that young people in particular had higher AMD and lower BMI.

### 3.2. Portion Size Results

Table 1 shows the portion size for each food referenced in the survey [36]. According to the type of food, portion sizes of XXS, XS, S, M, L, and XL were assigned and were adapted to the usual weight or volume for that food. Thus, Table 3 shows a summary of the mean results per country found in this study. In general, Spanish participants tend to have a higher average consumption of several food categories compared with the German participants. For instance, Spaniards showed higher average consumption of rice, meat, legumes, and fruit portions. On the other hand, Germans exhibited higher consumption of pizza, lasagne, and stews. Both countries had similar consumption levels for beverages, raw vegetables, breakfast cereals, (green) salads, cooked vegetables, fruits, pasta, fish, soups, and cakes. These results reflect differences in dietary preferences and habits between the populations of Germany and Spain (Wilks’ Lambda = 0.478, *p* < 0.01), indicating that certain foods are more popular or consumed in larger quantities in one country compared to the other.

**Figure 3:**
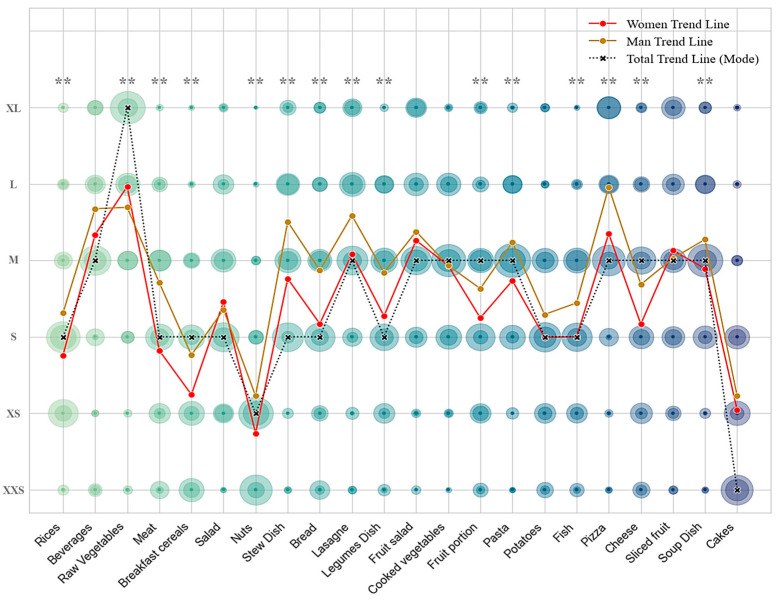
Differences in food size choices by gender and most preferred sizes. Circle size is proportional to frequency. Differences between gender. ** Significant at the *p* < 0.05 level.

The gender distribution revealed a preponderance of female participants in both countries, with a particularly pronounced female representation in Germany. The findings indicated a significant impact of gender on the selection of food portion sizes in total (Wilks’ Lambda = 0.704, *p* < 0.01) and across different food groups. Figure 3 shows the most frequently selected trends for each group and a mean of the trends by gender, highlighting significant differences. In general, men selected larger portion sizes than women, with the exception of raw vegetables.

In addition, other factors were analysed, but no clear significant differences were found, such as by type of employment or education level.

## 4. Discussion

### 4.1. Dietary Behaviour of the Subjects and Comparative Portion Size Estimation

The present study examined the influence of individual differences on food portions selection. These discrepancies can be largely attributed to the presence of similar or distinct attributes within the Spanish and German populations across multiple dimensions, as illustrated in Table 2.

The results obtained demonstrate statistically significant differences between the two genders (Figure 3). This difference may be due to men having a higher energy demand than women, or to the fact that for some food groups such as meat, these women consume less but consume more vegetables than men [37]. In addition, women were more frequently able to estimate the correct portion size from images than men [29], which is in line with other studies were men had more difficulties estimating the correct portion size [38].

The cohort from Spain was significantly younger (average age of 26.3 years) than that from Germany (average age of 40.8 years), which may have influenced other variables such as habits and living conditions [39]. The results indicated significant variations in the selection of food portion sizes depending on the consumer’s age. This may be because younger people consume more ultra-processed foods [40], or that young people have a greater ability to estimate portion size than older adults [39].

Table 2 illustrates the differences in body mass index (BMI) between the two groups. While both groups had an average BMI within the normal range, the German cohort exhibited a slightly higher BMI than the Spanish cohort. The statistically significant differences observed in BMI values suggest that there are variations that may be influenced, and this should be taken into account when evaluating the choice of food portion size [38]. Studies suggest that factors such as BMI, the perceived healthiness, or the energy density of the product may play a significant role. For example, foods that are perceived to be satiating were chosen in larger portions, whereas in some cases foods that are perceived to be more energy-dense were chosen in smaller portions, in line with the idea that portion size is important for weight maintenance or even loss [38]. The results of this study are consistent with the above, showing that in some cases BMI is a relevant factor in the selection of some food groups, such as vegetables. Furthermore, it has been demonstrated that factors such as low income and rural origin have a significant impact on disparities. However, these factors do not attain statistical significance in the present study. In these cases, food photographs presented in standardised portion sizes are essential for a correct estimation [41].

**Figure 4:**
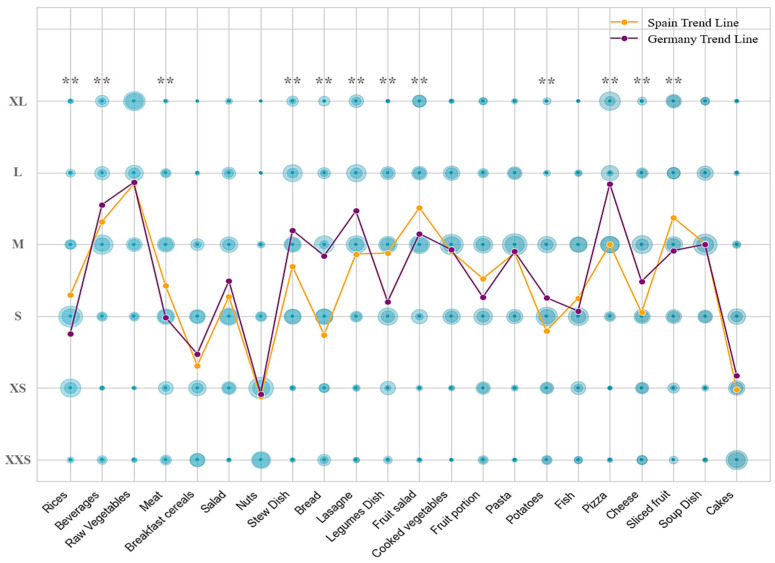
Differences in food size choices by country. Circle size is proportional to frequency. Differences between country. ** Significant at the *p* < 0.05 level.

As shown in Table 3, a comparison of food consumption between German and Spanish groups revealed several statistically significant differences in portion sizes for various food items. In Germany, the average consumption of rice was (127.43 ± 60.96 g), while in Spain it was higher (165.42 ± 83.85 g). Similarly, meat consumption was higher in the Spanish cohort (202.90 ± 81.65 g) compared to the German one (175.66 ± 75.77 g). Notably, Germans had a statistically significant higher portion size for stews (372.79 ± 127.04 g) and lasagne (402.66 ± 89.62 g) than Spaniards (318.64 ± 123.26 g) and (350.80 ± 112.04 g), respectively. On the other hand, Spanish participants had a higher intake of legumes (314.88 ± 112.03 g) compared to Germans (243.48 ± 105.75 g). Additionally, Germans ate significantly larger portions of pizza (481.39 ± 174.19 g) compared to Spaniards (353.23 ± 171.75 g), and similarly for sliced fruit (160.14 ± 48.72 g) in Germany vs. (176.91 ± 33.36 g) in Spain. These differences highlight varying dietary habits between the two countries, with some foods consumed at larger quantities in one country than in the other. These disparities may be influenced by cultural preferences, dietary guidelines, and lifestyle factors specific to each country [42]. Considering the differences in food choices between countries (Figure 4), the results showed that German subjects chose larger portion sizes for dishes such as stews and potatoes, which could be explained by their traditional food consumption [31].

The German population also tended to consume more ultra-processed products, which could explain why larger sizes of foods such as lasagne or pizza are chosen [43]. In contrast, individuals in Spain tended to consume larger portions of fruits and vegetables, which may be attributed to their adherence to a Mediterranean dietary pattern [42]. Consistent with other studies, the Spanish cohort exhibited significantly higher adherence to the Mediterranean diet compared to the German participants (Figure 1) [42]. Significant differences were observed in populations with high adherence to the Mediterranean diet in terms of larger portion sizes for foods such as various fruits and legumes, which can be attributed to the fact that these foods constitute the fundamental elements of the Mediterranean diet [22]. In terms of dietary habits, omnivores comprise the majority of the participants, with frequent use of spices, especially in Germany. Bread and dessert consumption varied, with bread consumption more frequent in Spain and non-dessert consumption more common in Germany. Water was the preferred beverage in both countries, with notable consumption of sparkling water in Germany. The results of this selection are indicative of a cultural phenomenon and an evolution of dietary habits [31].

In general, intermediate portion sizes were the most frequently chosen, which were designed to align with typical consumption sizes [23,27,29]. This trend was not followed by raw vegetables, nuts, and cakes (Figure 3). This is consistent with validation studies of the photographic atlas, where participants selected the image of the correct or adjacent portion size [29]. This confirms that the assigned average portion sizes and images were appropriate, except for some of the foods.

Possible explanations exist for why some groups did not follow the trend. For instance, smaller portion sizes are often overestimated [29], or there is often a decrease in portion sizes of unhealthy foods [44]. For example, this was reflected in cakes or pies, where poor concordance results were obtained, which could justify the findings of the present results [29]. Although some studies claim that plate size does not affect estimation, in some cases, when the large plate size was used, subjects increased their vegetable portion [38]. Furthermore, external factors have been demonstrated to exert influence over the selection and consumption of food portions. Such factors include the price-quality ratio, mindless eating, and biases. To illustrate this point, the bias of considering a food as a unit can lead to consuming a portion regardless of food size. Similarly, dividing food into smaller units has been found to reduce the amount of food that people could eat [38].

In recent years, there has been a global increase in the size of food portions, particularly in high-calorie foods. This phenomenon highlights a certain complicity on the part of the food industry [38]. This trend is also visible in home-cooked meals, as evidenced by the evolution of cookbooks and restaurant menus [38]. In the context of food portion classification, the terms ‘XXL’ and ‘S’ typically refer to extra-large and small portion sizes, respectively. In the food sector, these portion classifications are frequently employed to standardise portion sizes and assist consumers in selecting more practical portion sizes [38,45]. For example, in the context of fast food or restaurant meals, this is frequently observed [38,45]. Therefore, the same methodology as used in the Vietnamese atlas was chosen, allowing recipes to be tabulated with portion sizes for each meal: (XXS–XL). This study involved six portions, because research has shown that using an impartial number of portions can cause respondents to be biased towards the intermediate portion [33]. The results suggest that, in general, the tendencies of each subject tend to be similar and that people who choose large sizes will choose most products. This may help to create a tool that provides appropriate sizes for each subject. Smaller sizes were chosen as the study focuses on healthy nutrition and larger sizes are not recommended. Additionally, smaller sizes could be useful for extrapolation to the child population [32,35].

### 4.2. Essential Features to Enhance the Usability and Accuracy of the i-Diet App

The application of new technologies has resulted in notable advancements in dietary research, demonstrating that their efficacy is comparable or superior to that of traditional methods [12]. In recent years, mobile applications have become a useful tool for recording dietary intake. Speak4Diet is an example of such an application, which uses artificial intelligence to analyse and track dietary habits. However, mobile app-based dietary monitoring and recommendations have limitations. Users may find it tedious and adherence might be low [11]. Considering food preferences in a personalised nutrition app becomes essential. The study’s findings will contribute to the development of modules and tools designed to enhance the user experience in terms of personalisation, with a particular emphasis on portion size.

Based on the results obtained, being able to choose the bread and beverage consumption and choice separately on the menu proposed by the i-Diet app could help increase dietary adherence [20]. The same could be applied to the choice of one or more dishes, or the decision to eat a dessert or not. Depending on the country or other factors, the decisions may vary, so offering free choice to the user will play a key role in the use of a personalised nutrition app, contributing to the user’s empowerment and autonomy [46]. In the culinary context, spices have been observed to be utilised in a variety of ways, suggesting a potential for their incorporation into novel culinary preparations. However, it should be noted that recipes are typically predetermined, thereby diminishing their relevance in relation to other variables in the context of a more precise personalisation. Therefore, specific modules or shortcuts were incorporated into the app to take into account user behaviour and offer a more personalised experience.

Considering allergies and intolerances is critical due to their potential adverse effects; thus, in addition to the commonly reported allergens, an “allergies” category was also added to i-Diet to allow the user to exclude other foods from the menu generation process [20]. In view of the findings, it is recommended that specific categories, such as additives, LTP, or further well-known, controversially discussed compounds, be introduced to facilitate the process for users.

The Mediterranean diet was proposed as a basis due to its health properties and the results obtained. Usually, Spanish people have a higher adherence to the Mediterranean diet, but in Germany there were people who used olive oil frequently and adopted certain Mediterranean eating habits. Consequently, the application was developed on the basis of this fundamental dietary model, proposing culinary creations that would enhance adherence to the Mediterranean diet while preserving the distinct characteristics of the country in which the application is implemented.

The study results allowed for a comparison with estimated typical food consumption and provided a more precise average range for classifying portion sizes for each subject. The study results showed that some food groups, such as cakes, vegetables, and nuts, did not meet the expected average portion sizes. Additionally, in some groups, there were clear differences between countries, ages, and genders. In fact, the simple indication of portion sizes, widely used in other food concepts, showed positive results and the use of XXS–XL sizes now consolidated in the i-Diet app [20]. This information will help to minimise portion size issues associated with the use of the app. Furthermore, the results facilitated the integration of a module into the project’s app, enabling subjects to select their portion size range (from S to XL, including food photographs from the previously validated photographic food atlas) more quickly and accurately. This should help to improve future dietary recommendations based on these portion size ranges.

### 4.3. Study Limitations

The present study is subject to some limitations that must be taken into consideration. Firstly, the sample, although composed of 224 participants, is not designed to establish generalisations about the findings for both countries; rather, it is intended to be used as a starting point for the development of more precise nutrition strategies. Secondly, despite efforts to make the population groups as homogeneous as possible, it only collected responses from a limited group of people, and there are demographic differences between the two groups, with the Spanish sample having an average age that is notably younger. Furthermore, it is important to note that additional factors, such as physiological status and economic viability, may also influence dietary intake and portion sizes. However, the present study is specifically focused on the influence of geographic location, gender, age, and weight on dietary habits. The employment of an online questionnaire, though widely utilised and validated, is susceptible to self-report bias. The visual perception of food portions is subject to variation, and the methodology provides only an instantaneous assessment of eating habits, lacking longitudinal follow-up.

## 5. Conclusions

Significant differences in dietary behaviour and portion size selection between the studied populations of Spain and Germany were observed. The Spanish cohort was younger and showed a higher incidence of other intolerances, whereas the German cohort had a higher employment rate and more married individuals. In both countries, most people were omnivores, although bread and dessert consumption were more common in Spanish participants. The Mediterranean diet was mostly followed in the Spanish group, reflecting cultural differences in eating habits. The body mass index (BMI) was slightly higher in Germans, although it was within normal ranges. Portion size comparisons revealed significant differences in the consumption of various foods between the two countries. The Spanish participants consumed more rice, meat, and legumes, while Germans had larger portions of stews, lasagne, and pizza. These differences highlight distinct dietary habits influenced by cultural preferences and dietary guidelines. The findings of this study demonstrate the potential for the development of personalised nutrition apps that take into account user preferences, with the objective of increasing dietary adherence. Consequently, this will facilitate more accurate dietary recommendations and improve health outcomes. The study provided detailed information on the dietary habits and preferences of the adult population in Spain and Germany, which has enabled the optimisation of the i-Diet app. The findings of this study have enabled the i-Diet app to offer users greater choice and empower them in their decision-making processes, thereby facilitating improved adherence to the dietary recommendations set out in the project’s intervention study. Therefore, this methodology could be useful in a general context, reflecting the diversity of people who could use a personalised nutrition app.

## Figures and Tables

**Table 1:** Food categories and the range of portion sizes.

Type of Food		XXS	XS	S	M	L	XL
	Portion Range						
Rices (g)		39	85	131	224	316	362
Beverages (mL)		100	200	300	400	500	600
Raw vegetables (g)		32	49	67	101	136	153
Meat (g)		56	108	168	246	308	400
Breakfast cereals (g)		16	23	31	45	60	67
Salad (g)		88	150	213	338	463	525
Nuts (g)		15	49	82	116	183	250
Stew dish (g)		93	158	222	352	481	546
Bread (g)		22	34	48	63	94	126
Lasagne (g)		135	187	238	341	444	496
Legumes dish (g)		80	140	200	320	440	500
Fruit salad (g)		39	64	89	139	189	214
Cooked vegetables (g)		26	56	85	145	204	234
Fruit portion (g)		53	83	166	266	376	532
Pasta (g)		55	108	161	266	372	425
Potatoes (g)		61	97	131	204	275	311
Fish (g)		43	58	82	245	345	560
Pizza (g)		42	84	168	335	502	670
Cheese (g)		13	25	36	60	83	95
Sliced fruit (g)		28	56	83	139	194	222
Soup dish (mL)		33	89	145	257	369	425
Cakes (g)		67	83	98	129	160	176

**Table 2:** Characteristics of the surveyed populations.

Feature		Spain	Germany	Total
Population		84	140	224
Age (years)	Average ± SD	26.3 ± 10.2	40.8 ± 14.9	35.4 ± 15.1
Gender	Male	31	46	77
	Female	53	94	147
Origin	Asian	0	1	1
	European	80	138	218
	Latin	4	1	5
Allergies and Intolerances	No	64	116	180
	Nuts	0	8	8
	Dairy	5	9	14
	Others	15	3	18
Education	Primary	4	3	7
	Secondary	10	25	35
	Higher	70	112	182
Employment	Unemployed	1	1	2
	Employed	18	90	108
	working part-time	8	18	26
	Studying	55	26	81
	Retired	2	5	7
Marital status	Married	6	54	60
	Divorced	0	6	6
	Other	4	36	40
	Single	74	44	118
Share home	With friends	25	16	41
	With family	43	41	84
	With partner	8	55	63
	Other	1	0	1
	Alone	7	28	35
Housing	Rented	36	75	111
	Owned	34	60	94
	Other	14	5	19
Living in	Urban areas	66	102	168
	Rural areas	18	38	56
Eating habits	Vegetarians	0	23	23
	Omnivores	98	103	201
Dietary behaviour	Eat in company	61	100	161
	Alone	23	40	63
Use spices	Sometimes	14	3	17
	Never	5	1	6
	Always	65	136	201
Dishes during meals	Other	5	6	11
	Single dish	61	128	189
	First and second dish	18	6	24
Eat dessert	Sometimes	18	63	81
	Never	20	66	86
	Always	46	11	57
Bread consumption	Sometimes	24	43	67
	Never	28	71	99
	Always	32	26	58
Drink during meal	Water	69	75	144
	Sparkling water	1	41	42
	Beer/Wine	4	3	7
	Never	6	6	12
	Other	0	5	5
	Soft drink	4	2	6
	Juices	0	8	8
AMD	Average ± SD	9.63 ± 1.72	6.09 ± 1.99	7.42 ± 2.56
Physical activity	Intense (>5 times/week)	13	17	30
	Light (walking)	26	48	74
	Moderate (3 times/week)	39	57	96
	Very intense (2 h/day)	4	1	5
	Very light	2	17	19
BMI (kg/m^2^)	Average ± SD	23.07 ± 3.92	24.23 ± 4.43	23.79 ± 4.27

**Table 3:** Average food consumption by country according to surveys.

Food	Country	Average ± SD	Portion Size Group	*p* < 0.05 *
Rices (g)	Germany	127.43 ± 60.96	S	**0.0003**
	Spain	165.42 ± 83.85	M	
Beverages (mL)	Germany	355.40 ± 138.40	M	0.4067
	Spain	328.57 ± 193.62	M	
Raw vegetables (g)	Germany	121.36 ± 33.38	L	0.7752
	Spain	121.19 ± 35.53	L	
Meat (g)	Germany	175.66 ± 75.77	M	**0.0123**
	Spain	202.90± 81.65	M	
Breakfast cereals (g)	Germany	29.30 ± 11.12	S	0.2453
	Spain	27.92 ± 12.64	S	
Salad (g)	Germany	287.36 ± 119.60	M	0.1556
	Spain	264.88 ± 109.29	M	
Nuts (g)	Germany	52.17 ± 39.51	S	0.4533
	Spain	52.38 ± 46.91	S	
Stew dish (g)	Germany	372.79 ± 127.04	L	**0.0021**
	Spain	318.64 ± 123.26	M	
Bread (g)	Germany	62.43 ± 34.18	M	**0.0001**
	Spain	35.12 ± 27.52	S	
Lasagne (g)	Germany	402.66 ± 89.62	L	**0.0005**
	Spain	350.80 ± 112.04	L	
Legumes dish (g)	Germany	243.48 ± 105.75	M	**0.0001**
	Spain	314.88 ± 112.03	L	
Fruit salad (g)	Germany	147.93 ± 47.10	M	**0.0198**
	Spain	163.43 ± 42.77	M	
Cooked vegetables (g)	Germany	141.55 ± 48.20	L	0.8939
	Spain	142.07 ± 49.35	L	
Fruit portion (g)	Germany	284.60 ± 165.06	L	0.1463
	Spain	315.48 ± 176.33	L	
Pasta (g)	Germany	227.11 ± 76.05	M	0.8626
	Spain	229.70 ± 76.24	M	
Potatoes (g)	Germany	159.04 ± 65.14	M	**0.0033**
	Spain	135.62 ± 51.37	M	
Fish (g)	Germany	136.83 ± 94.33	M	0.1700
	Spain	164.94 ± 114.80	M	
Pizza (g)	Germany	481.39 ± 174.19	L	**0.0001**
	Spain	353.23 ± 171.75	M	
Cheese (g)	Germany	49.68 ± 22.53	M	**0.0258**
	Spain	42.76 ± 21.83	M	
Sliced fruit (g)	Germany	160.14 ± 48.72	M	**0.0288**
	Spain	176.91 ± 33.36	L	
Soup dish (mL)	Germany	296.36 ± 86.22	L	0.9097
	Spain	297.63 ± 90.79	L	
Cakes (g)	Germany	102.46 ± 31.02	M	0.2586
	Spain	97.30 ± 29.55	M	

* Bold numbers denote statistically significant differences.

## Data Availability

Data are available on request.
